# Supplementary figures and images for: White matter hyperintensities and the risk of vascular dementia: a systematic review and meta-analysis
Source: PeerJ. 2025 Jun 16;13:e19460. doi: 10.7717/peerj.19460 (PMC12178243; doi:10.7717/peerj.19460)

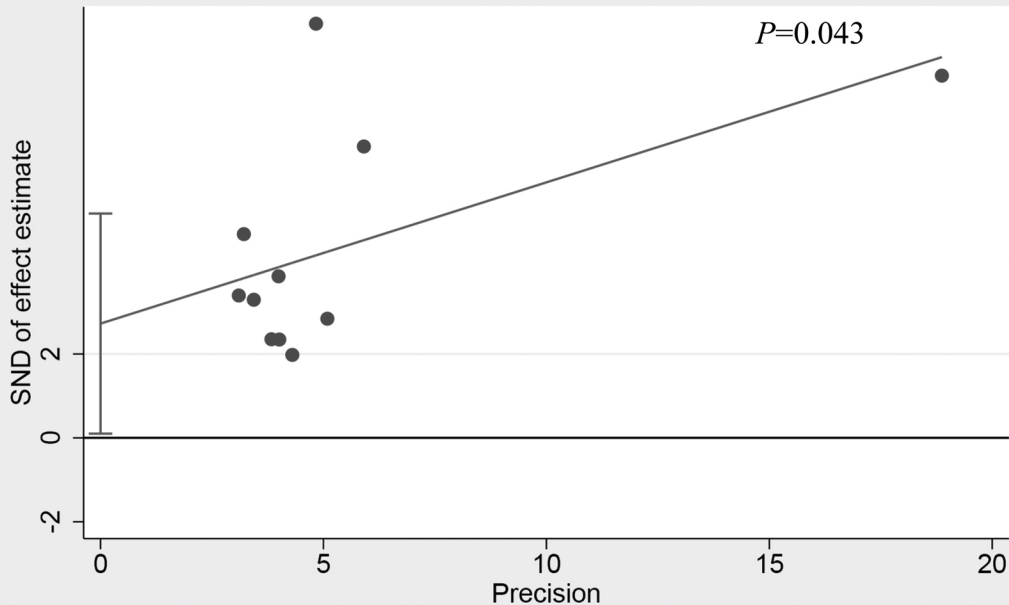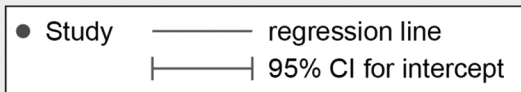

Supplement: Supplemental Information 2 [file peerj-13-19460-s002.pdf]

Filled funnel plot with pseudo 95% confidence limits

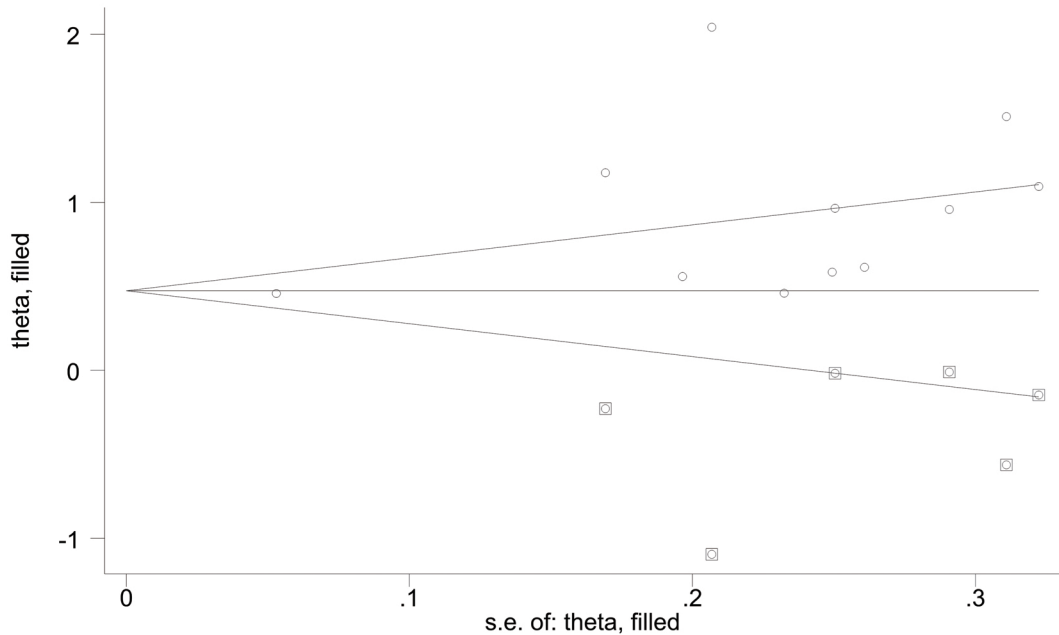

Supplement: Supplemental Information 3 [file peerj-13-19460-s003.pdf]
