# Supplementary material for: White matter hyperintensities and the risk of vascular dementia: a systematic review and meta-analysis
Source: PeerJ. 2025 Jun 16;13:e19460. doi: 10.7717/peerj.19460 (PMC12178243; doi:10.7717/peerj.19460)
Supplement: Supplemental Information 4 [file peerj-13-19460-s004.docx]

**Systematic Review/Meta-Analysis Search**

- Please name the two authors who performed the Search Strategy in the methods section of your manuscript.

Reply: The search strategy and data extraction were independently conducted by Wei Luo and Zhiqiang Dai.

- You must also describe how disagreements were resolved, and identify the referee.

Reply: Any disagreements that arose during the search and selection process were discussed between Wei Luo and Zhiqiang Dai. Final decisions were made by the third-party corresponding author, Dr. Yang Zhang, who served as the referee.

**Systematic Review and/or Meta-Analysis Rationale**

For systematic reviews / meta-analyses, authors need to provide the following information:

- describe ***the audience it is intended for***

Reply: The intended audience for this article includes medical professionals such as neurologists, geriatricians, and radiologists, as well as researchers in the fields of neurology, vascular medicine, and geriatric health. Additionally, it is relevant to public health policymakers and healthcare providers who are involved in the management and prevention of dementia and related neurological conditions. The study's findings on the relationship between white matter hyperintensities (WMH) and vascular dementia (VaD) can also be of interest to medical students and trainees who are learning about neurodegenerative diseases.

- Please [upload your answers in a supplemental file (File type 'Other')](https://peerj.com/manuscripts/111926/files/).

Reply: Yes, we have uploaded our answers in a supplemental file with the file type 'Other'.
